# Paraneoplastic dermatomyositis revealing undifferentiated nasopharyngeal carcinoma at early stage: a case report

**DOI:** 10.1186/s13256-021-03154-0

**Published:** 2022-01-04

**Authors:** Souleymane Panandtigri, Nioka Pierre Xavier Siaˡ, Meryeme Charkaouiˡ, Nadia Benchakrounˡ, Zineb Bouchbikaˡ, Hassan Jouhadiˡ, Nezha Tawfiqˡ, Souha Sahraouiˡ, Abdellatif Beniderˡ

**Affiliations:** Center Mohamed VI for the Treatment of Cancers of the University Hospital Center IBN ROCHD Casablanca, Casablanca, Morocco

**Keywords:** Dermatomyositis, Paraneoplastic, Nasopharynx, Corticosteroid therapy, Radiotherapy

## Abstract

**Context:**

Dermatomyositis is a rare autoimmune disease characterized by noninfectious inflammatory damage of skin and predominant muscles in the belts. It is believed to be associated with about 1 in 1000 cases of nasopharyngeal carcinoma. This association has been described for locally advanced stages II and III nasopharyngeal carcinoma. It has rarely been described in the early stages (stage I).

**Case presentation:**

A 65-year-old Moroccan patient residing in Casablanca, with no particular history was referred to the Mohamed VI Center for the treatment of cancers of the University Hospital Center IBN ROCHD in Casablanca, for treatment of nasopharyngeal cancer. He was admitted in poor general condition, performance status 3, with erythema on the face, neck, and extremities. The diagnosis of paraneoplastic dermatomyositis was made owing to progressive muscle weakness and elevation of muscle enzymes associated with the typical rash of the face and hands. He received corticosteroid therapy and then radiotherapy to the nasopharynx with good clinical outcome, disappearance of skin lesions, and recovery of muscle strength.

**Conclusions:**

We report this case of dermatomyositis in early-stage nasopharyngeal carcinoma, which is a rarely described entity. Rapid treatment of dermatomyositis improved the patient’s quality of life and enabled him to support specific cancer treatments. This can be used as an element of early diagnosis and monitoring after treatment.

## Introduction

Dermatomyositis (DM) is a rare autoimmune disease characterized by noninfectious inflammatory damage of skin and predominant muscles in the belts [1]. It is associated with a malignant tumor in 18–32% of cases, and may be indicative of cancer, including ovarian, bronchial, breast, and head and neck cancers, as well as lymphomas more rarely. It can appear simultaneously or after diagnosis [2]. The incidence of dermatomyositis associated with nasopharyngeal carcinoma (NPC) is 1 in 1000 cases [3]. This association has been described for locally advanced stages II [3] and III [4].

The objective of this study is to report a case of undifferentiated carcinoma of the nasopharynx (UCNT) at an early stage (stage I). We describe the diagnostic, therapeutic, and prognostic aspects of this entity, although rare. Thus, clinicians will be able to make the diagnosis at early stages.

## Case presentation

**Fig. 1:**
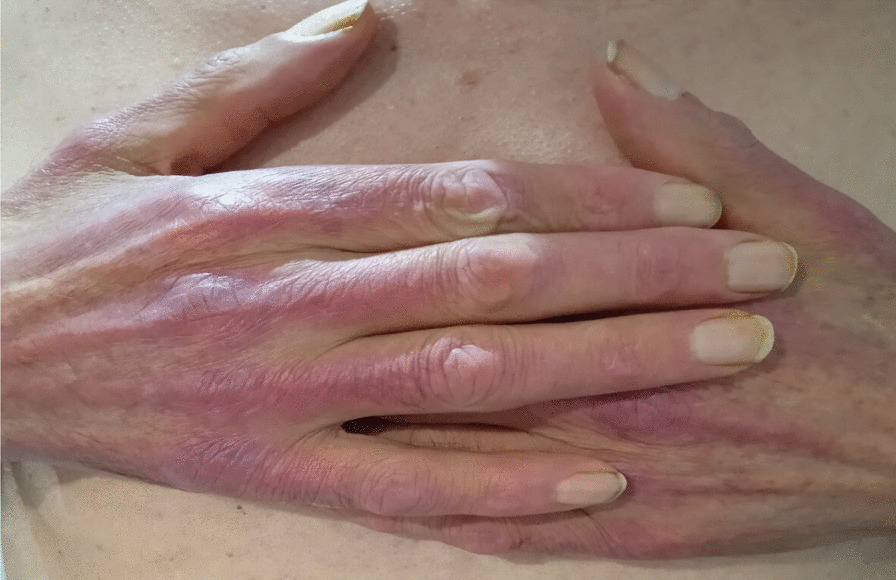
Erythematous rash on the hands

**Fig. 2:**
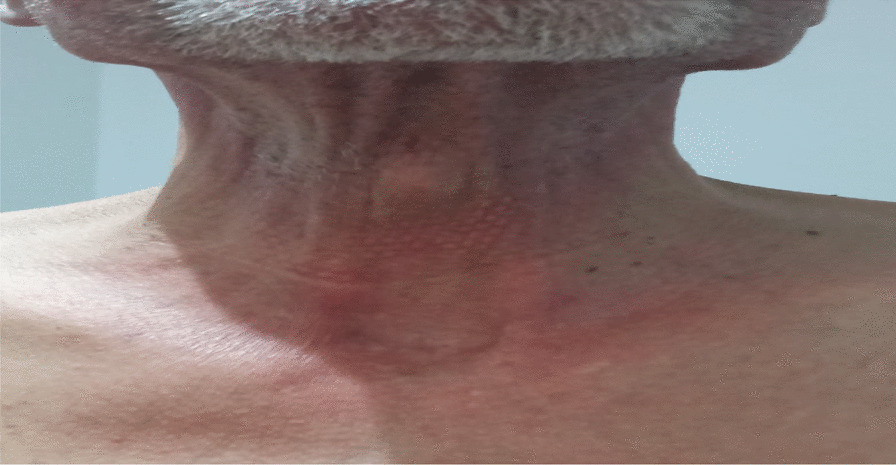
Erythematous rash on front of neck

**Fig. 3:**
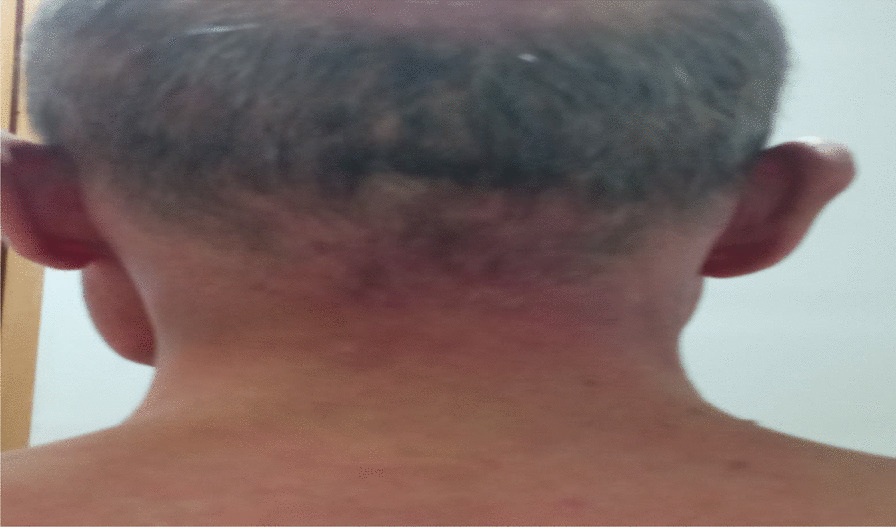
Erythematous rash on posterior side of neck

A 65-year-old Moroccan origin patient with no particular history was referred to the Mohamed VI Center for the Treatment of Cancers of the University Hospital Center (CHU) IBN ROCHD, Casablanca. The onset of his symptomatology dates back 3 months to the onset of intermittent solid dysphagia (a symptom of dermatomyositis because muscle damage can lead to swallowing disorders), which became total, motivating establishment of a nasogastric feeding tube, and associated with a rash on the face and hands, with intense fatigue and nonquantified significant weight loss. On clinical examination, the patient was found to be highly impaired, with a performance status (PS) of 3 and a rash on the face, neck, and extremities with a Cutaneous Dermatomyositis Disease Area and Severity Index (CDASI) score of 15 (Figs. 1, 2 and 3). The examination also revealed a muscle deficit in the axial muscles rated at eight, according to manual muscle testing (MMT), proximal and distal muscles rated at 32 and 8, respectively, with an overall MMT score of 48 out of 260 [1]. In the paraclinical check-up for cancer diagnosis, panendoscopy showed a tumor process in the posterior wall of the nasopharynx. The biopsy found an undifferentiated carcinoma of the nasopharynx (UCNT), with immunohistochemical expression of p63. p63 is not a specific marker for the nasopharynx, but it is a general marker for squamous cell carcinomas. We have other markers such as p40 and LMP1, but only p63 was tested due to availability. Magnetic resonance imaging (MRI) of the nasopharynx and positron emission tomography (PET) also showed the tumor process at the posterior nasopharyngeal wall of the nasopharynx classified as stage I (T1N0M0).

Owing to clinical signs suggestive of dermatomyositis, a requested biological assessment showed increased lactate dehydrogenase (LDH) 301 IU/L, with normal creatine phosphokinase (CPK) 148 IU/L and antinuclear antibodies positive to 1/640 of homogeneous types. The final diagnosis of paraneoplastic dermatomyositis with an overall assessment of disease activity by the physician (IMACS) assessed on a four-point Likert scale corresponding to a very severe active disease associated with stage I nasopharynx UCNT. In view of the severity of the dermatomyositis, it was necessary to start rapid treatment because of its extreme activity [1].

The patient received corticotherapy of methylprednisolone (1 mg/kg/d) injectable for 3 days, then oral relay with 60 mg prednisolone for 4 months with a slow reduction of 2.5 mg/week.

**Fig. 4:**
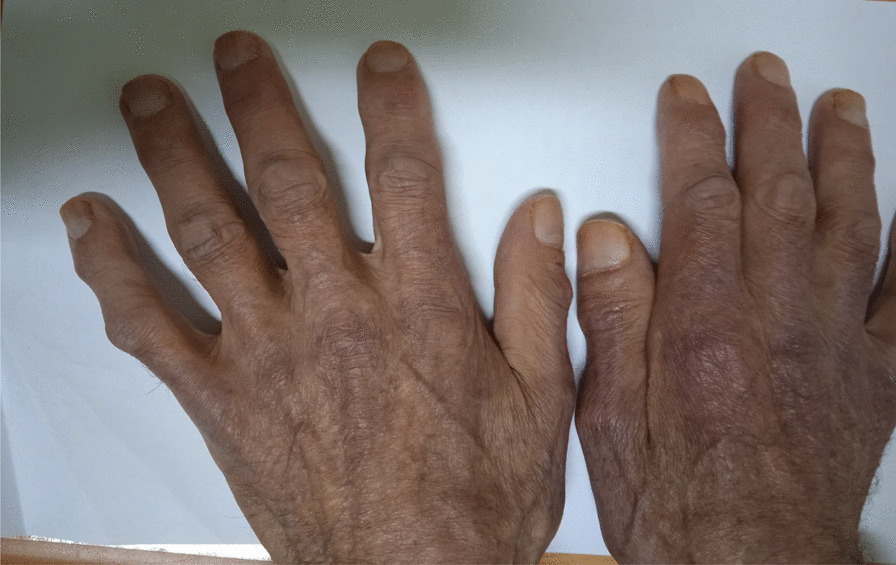
Disappearance of skin lesions

A significant improvement occurred with corticosteroid therapy: the patient’s performance status improved to 2, with removal of the nasogastric feeding tube and a marked regression of skin lesions to a CDASI score of 11. There was a decrease in motor deficit with an MMT of 208 and the muscle LDH reduced to 160 IU/L and CPK to 40 IU/L. The treatment of his cancer was then initiated, with exclusive radiotherapy at a rate of 70 grays (GY) with a total dose on the tumor of 2 GY/fraction over 35 sessions with five sessions/week. At the end of the etiological treatment, the patient was autonomous with a performance status of 1, skin lesions had disappeared (Fig. 4), and he had total recovery of muscle strength.

## Discussion

Nasopharyngeal carcinomas (NPCs) account for less than 1% of all malignant tumors and affect about 1 person in 100,000 in North America and Western Europe. It is more common in Asia and North Africa, where it affects 5–9 people per 100,000 [5]. The undifferentiated pathological subtype is the most common, most often associated with paraneoplastic syndromes such as fever, leukemoid reactions, and osteoathropathy [6]. The combination of dermatomyositis and cancer is common, affecting 18–32% of patients [2]. However, its association with pharyngeal carcinoma is not well described. Most often it is associated with the undifferentiated type, somewhat less frequently associated with the differentiated type, and never with well-differentiated cancer [7]. It may be more severe than the consequences of the primary tumor itself and may precede, follow, or be concomitant with the diagnosis of cancer. This is the finding in our case where symptomatology was dominated by dermatomyositis.

The pathogenesis of dermatomyositis is still poorly known, and several mechanisms have been suggested. Two theories have been developed: hormonal theory and immunological theory. Hormonal theory hypothesizes that it is the tumor that secretes biologically active hormonal polypeptides that are homeostatically inappropriate. These polypeptides are thought to be responsible for different clinical endocrine syndromes [7]. In the immunological theory, paraneoplastic syndrome is the result of cross-reactions of antibodies produced against tumor antigens, with normal tissues having a similarity of structures [7].

The diagnosis of dermatomyositis is based on five criteria according to Bohan and Peter [8]: progressive, symmetrical muscle weakness of the neck girdles and flexor muscles; dermatological signs (heliotropic rash with periorbital edema; Gottron's papules (scaly dermatitis on the joints of the fingers); dermatitis on the elbows, knees, and feet; and a muscle biopsy in favor of myositis.

An increase in serum muscle enzymes is indicative of muscle necrosis (especially CPK, aldolase, LDH). These are increased in 70–90% of patients, however a normal rate should not rule out the diagnosis [1]. An electromyographic profile in favor of muscle damage. The presence of three or four of these criteria, in addition to the rash, enables the diagnosis of dermatomyositis, and the presence of two criteria associated with the rash is very suggestive of dermatomyositis. Thus, faced with the progressive muscle weakness and elevation of LDH associated with the typical rash, we made the diagnosis of paraneoplastic dermatomyositis.

Dermatomyositis can be idiopathic, especially in children. However, it is usually linked to a malignant tumor, as shown in the epidemiological study by Hill *et al.*, in which 32% of dermatomyositis were associated with cancers of the ovary, lung, pancreas, breast, gastrointestinal tract, or non-Hodgkin lymphoma [2]. Also, Chan published a series of dermatomyositis cases in Singapore, in which 41% were nasopharyngeal carcinomas [9]. In addition,a statistical study carried out in China found an association of dermatomyositis with cancer of 20.3%, of which 78.5% concerned nasopharyngeal carcinoma [10].

The treatment of dermatomyositis in a cancer patient involves treatment of both the dermatomyositis and the tumor. Treatment is the same in patients with or without associated cancer and aims to increase muscle strength and improve extramuscular manifestations. First-line treatment is based on high-dose corticosteroid therapy with the usual hygienic-dietary measures of corticosteroid therapy [11]. Before the use of corticosteroids, the prognosis of dermatomyositis was poor, with mortality of 50–60% [12].

Corticosteroids are prescribed at a dosage of 1 mg/kg/d for 4–8 weeks on average, until the regression of clinical signs and the reduction or normalization of muscle enzymes. Then, a progressive reduction can be initiated until the minimum effective dose is achieved for 6–9 months without relapse [13]. For severe forms of dermatomyositis, the use of intravenous bolus methylprednisolone at a dosage of 1 mg/kg/d for 3 d, then oral relay is recommended [12]. This was the case for our patient admitted in an altered state who received an injectable bolus and then oral relay. However, in the case of refractory or corticosteroids, other treatment options are used such as methotrexate, azathioprine, intravenous immunoglobulins, and rituximab [14].

In addition to corticosteroid therapy, treatment for nasopharyngeal carcinoma should be carried out. In stage I patients, treatment is exclusive radiotherapy of 70 GY at 2 GY/fraction over 35 sessions (5/week) as in our case.

The prognosis of nasopharyngeal carcinoma with dermatomyositis is the same as that of a nasopharyngeal carcinoma in general [10]. In addition, many authors have reported that a complete remission after radiation therapy on the tumor results in the disappearance or improvement of the symptoms and physical signs of dermatomyositis. Relapse of dermatomyositis was correlated with locoregional recurrence or the detection of metastases, therefore, a monitoring element after treatment of nasopharyngeal carcinoma should be undertaken.

## Conclusion

Nasopharyngeal carcinoma with paraneoplastic dermatomyositis is a rare but specific entity. The diagnosis of dermatomyositis is based on five clinical and paraclinical criteria. It is sometimes indicative of some nasopharyngeal carcinomas, which is an element of both early diagnosis and surveillance. Thus, in the face of dermatomyositis in patients from endemic areas, nasopharyngeal carcinoma should be investigated as a priority. In addition, the association of dermatomyositis with nasopharyngeal carcinoma does not appear to influence the prognosis of cancer, hence the value of rapid management of dermatomyositis to improve the patient’s quality of life and enable them to support specific cancer treatments.

## Data Availability

No object.

## Abbreviations

DM: Dermatomyositis
NPC: Nasopharynx carcinoma
UCNT: Undifferentiated carcinoma of the nasopharynx
CHU: University Hospital Center
PS: Performance status
CDASI: Cutaneous Dermatomyositis Disease Area and Severity Index
MMT: Manual muscle testing
PET: Positron emission tomography
MRI: Magnetic resonance imaging
IMACS: Global assessment of disease activity
LDH: Lactate dehydrogenase
CPK: Creatine phosphokinase
Gy: Grays
Fr: Fraction
